# Increased secretion of salivary glands produced by facial vibrotactile stimulation

**DOI:** 10.1080/08990220802611649

**Published:** 2008-11-26

**Authors:** Hisao Hiraba, Masaru Yamaoka, Mika Fukano, Tadao Fujiwara, Kouichirou Ueda

**Affiliations:** 1 Department of Dysphasia Rehabilitation, Division of Functional Morphology Dental Research Center, Tokyo, Japan; 2 Department of Physics, Division of Functional Morphology Dental Research Center, Tokyo, Japan

**Keywords:** PWM circuit, vibration stimuli, headphone headset, masseter muscle belly, electric toothbrush

## Abstract

Patients with low-back pain can be evaluated immediately by means of an electrical tool that produces bony vibration to the lumbar spinal processes (Yrjama M, Vanharanta H. Bony vibrotactile stimulation: A new, non-invasive method for examining intradiscal pain. European Spine Journal 1994;3:233–235). In the rehabilitation of masticatory disturbance and dysphagia, an electric toothbrush is commonly used as an oral motor exercise tool for the facilitation of blood flow and metabolism in the orofacial region in Japanese hospitals. However, subjects receiving vibration in the facial regions reported increased salivary secretion. We attempted to develop an oral motor exercise apparatus modified by a headphone headset that was fixed and could be used for extended periods. The vibration apparatus of the heating conductor is protected by the polyethyle methacrylate (dental mucosa protective material), and electric motors for vibration control of the PWM circuit. We examined the amount of salivation during vibration stimuli on the bilateral masseter muscle belly, using a cotton roll positioned at the opening of the secretory duct for 3 min. Although the quantity of salivation in each subject showed various and large fluctuations in the right and left sides of the parotid and submandibular and sublingual glands, one or more of the salivary glands were effectively stimulated by 89 Hz vibration. The reported apparatus will be useful as an additional method in orofacial rehabilitation.

## Introduction

For the rehabilitation of masticatory disturbance and dysphagia, the Japanese guidebook of dental care for elderly people (Koureishya Shika Guidebook) states that the vibration of an electric toothbrush can be used as an oral motor exercise tool for the facilitation of blood flow and metabolism in the orofacial region (Ueda 2005). Furthermore, Burdette and Gale (1988) reported that tonic masticatory muscle activity might be effective in the treatment of myofascial pain-dysfunction patients. Although patients are comfortable using an electric toothbrush with their doctor, due to infirmity they are unable to maintain use on the affected part for long periods, and cannot adjust the frequency required for effective treatment, since patients often ask for a low frequency. We attempted to develop a new vibro-tactile stimulation apparatus that can be maintained on the affected part for long periods with changeable vibration frequency. After using the apparatus with subjects, they informally reported an increase in salivation, which was not the primary intended function of the device.

Salivation is produced by intraoral cavity stimuli such as food, acid taste and so on (Bridges 1981). It is known to be produced by salivary glands controlled by the reflex arc of parasympathetic nerves through trigeminal (somatosensory) or facial (taste) fibres related to the mechanoreceptors in the oral cavity or chemical-receptors in taste stimuli (Bridges 1981; Iversen et al. 2000). We were interested in experimentally verifying, and explaining, the informal reports of increased salivation with vibration applied to the face.

## Methods and materials

### Development of vibrotactile stimulation apparatus

**Figure 1:**
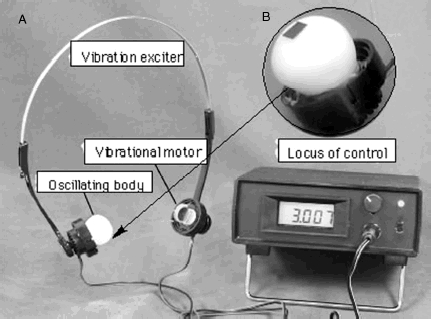
Apparatus for vibrotactail stimulation. (a) Vibrotactail exciter, vibrotactailal motor, oscillating body and locus of control. (b) Expanded oscillating body.

The vibrotactile stimulation apparatus consists of an oscillating body and control unit, as shown in Figure 1(a). The oscillating body is composed of the headphone headset equipped with vibrators as a substitute for positions of the bilateral microphones (Figure 1a). Vibrators utilized the vibration electric motor (VEM) (Rekishin Japan Co., LE12AOG). The VEM was covered in silicon rubber (polyethyl methacrylate, dental mucosa protective material, Shyofu Co.) for conglobating the stimulation parts and preventing the warming of the VEM’s temperature produced by the vibration of long periods, as shown in Figure 1(b).

**Figure 2:**
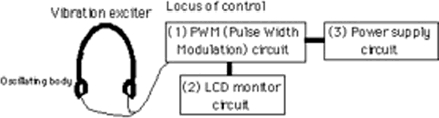
Design chart in locus of control.

**Figure 3:**
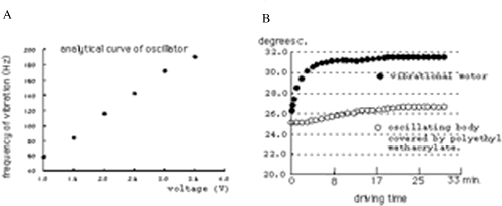
Analytical curve of oscillator (a) and changes in temperature of the vibrotactailal motor and oscillating body (b).

The control unit consists of three parts, the pulse width modulation (PWM) circuit, LCD monitor circuit and power supply circuit, as shown in Figure 2 (Yamaoka et al. 2007). The control unit interfaced with a PWN electric motor, delivered vibration frequencies in the 60–182 Hz range. We examined changes in the temperature of the device of the electric motor covered by polyethyl methacrylate. A temperature increase of only 1.6°C was observed after operation of the device for 30 min with an ambient room temperature of 25.3°C, as shown in Figure 3(b). This increase was the same at all operation frequencies.

### Normal subjects and quantities of salivation

We first explained in objects of experiments and recruited subjects understood by the informed consent. We measured the resting saliva firstly and stimulated saliva with 2% tartaric acid secondly in 39 subjects (male 30 and female 9), using measuring the amount of saliva absorbed in 3 min by a cotton roll placed at the opening of the secretory duct. We used cotton rolls with standardized 3 cm major axis and 1 cm in diameter. We first measured the weight of empty cotton rolls. Then the weight of cotton rolls soaked with saliva produced after 3 min was subtracted from that of empty (dry or pre-absorption) rolls. Cotton rolls were seated at the site of the buccal mucosa near the upper second molar tooth for the parotid gland and at the opening parts of elevation in the floor of the mouth for the submandibular and sublingual glands. The quantities of salivation from each gland in the right and left sides were measured for 3 min. First, we measured the resting saliva in each gland. After 10 min, we measured stimulated saliva with 2% tartaric acid. We put three drops on the tongue dorsum with a dropper from the bottle (about 0.3 ml).

Twenty-one healthy volunteers (14 male, 7 female) aged between 23 and 29 years were studied, after informed consent for the procedures. We examined the quantities of salivation was obtained during vibration stimuli on the bilateral masseter muscle belly for 3 min, using the cotton roll method. After that, we measured quantities of salivation for various stimuli defined by three frequencies (89 Hz = 1.5 V, 114 Hz = 2.0 V and 180 Hz = 3.3 V) with the interval time of 10 min. Measurements were collected in the afternoon (about 4 pm to 6 pm), and the examination was performed in a temperature-controlled room (about 21°C).

## Results

### Resting saliva and Stimulated saliva with 2% tartaric acid

First, we examined changes in quantity of salivation in each 3-min period of the resting saliva and stimulated saliva with 2% tartaric acid, using the cotton roll method in 39 subjects. In resting saliva, the quantities of salivation of parotid glands in the right and left sides are the same (15%, 6/39) or different (the larger values for the right side are 28%, 11/39, and those for the left side 56%, 22/39.), as shown in Panel A of Table I. Furthermore, different quantities are produced from submandibular and sublingual glands in the right side and those in the left side (the larger right side values are 38%, 15/39 and those for the left side 62%, 24/39), as shown in Panel A of Table I. On the other hand, in stimulated saliva with 2% tartaric acid, quantities of salivation of parotid glands in the right side and those in the left side are different (the larger values for the right side are 38%, 15/39 and those for the larger left side 62%, 24/39), as shown in Panel B of Table I. Furthermore, different quantities are produced from submandibular & sublingual glands in the right side and those in the left sides (the larger values for the right side are 46%, 18/39, and those for the left side 54%, 21/39), as shown in Panel B of Table I.

**Table 1:** Difference from the right and left sides in parotid or submandibular & sublingual gland.

*n* = 39	Right side is large	Left side is large	Right & left sides are same
Panel A: Resting saliva			
Parotid gland	28% (11)	56% (22)	15% (6)
Submandibular and sublingual glands	38% (15)	62% (24)	0
Panel B: Stimulating saliva with 2% tartaric acid			
Parotid gland	38% (15)	62% (24)	0
Submandibular and sublingual glands	46% (18)	54% (21)	0

**Figure 4:**
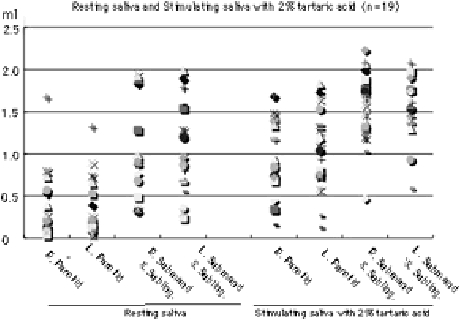
Resting saliva and stimulating saliva with 2% tartaric acid in each subject (n = 19).

**Figure 5:**
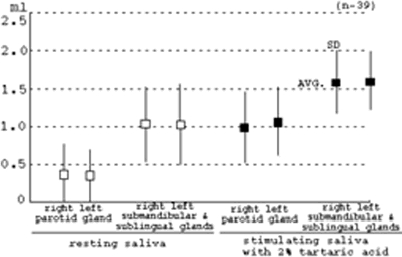
Averages and standard deviation of resting saliva and stimulating saliva with 2% tartaric acid in each gland.

However, in the resting saliva, maximum and minimum rates (3 min) in the parotid gland of the right side are 1.67 and 0.04 ml (Avg. = 0.37, SD = 0.39), and those in the left side are 1.32 and 0.04 ml (Avg. = 0.36, SD = 0.33). Maximum and minimum rates (3 min) in the submandibular and sublingual glands of the right side are 1.95 and 0.32 ml (Avg. = 1.03, SD = 0.49), and those in the left side are 1.99 and 0.24 ml (Avg. = 1.02, SD = 0.52). Furthermore, in stimulated saliva with 2% tartaric acid, maximum and minimum rates (3 min) in the parotid gland of the right side are 1.69 and 0.16 ml (Avg. = 0.99, SD = 0.46), and those in the left side are 1.83 and 0.13 ml (Avg. = 1.06, SD = 0.22). Maximum and minimum rates (3 min) in the submandibular and sublingual glands of the right side are 2.23 and 0.47 ml (Avg. = 1.58, SD = 0.42), and those in the left side are 2.08 and 0.59 ml (Avg. = 1.60, SD = 0.38), as shown in Figure 4. In particular, in the resting and stimulated saliva, the rate of averages (Avg.) and Standard deviations (SD) are almost the same quantities in the right and left sides of glands with the same name, as shown in Figure 5. However, the quantities of saliva in each gland in the subjects showed very large variations, even in the same glands, as shown in Figure 4.

### Stimulated saliva with vibrotactile stimulation

We obtained standard quantities of the resting saliva in each gland of each subject, and examined whether vibration stimuli had a greater effect in comparison with resting salivation.

**Figure 6:**
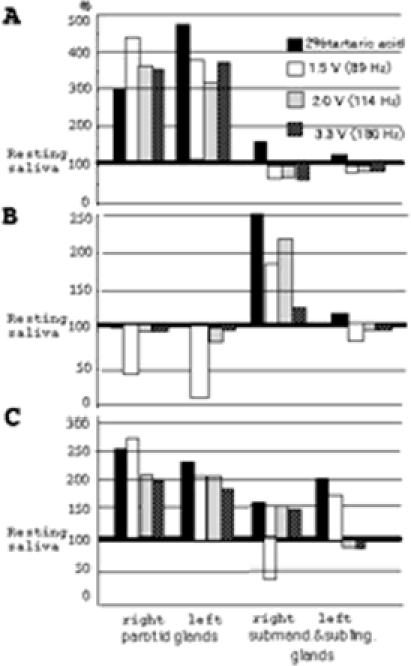
Examples of typical increased salivation patterns in the parotid and/or submandibular and sublingual glands. (a) Increased salivation in the parotid gland. (b) Increased salivation in the submandibular and sublingual glands. (c) Increased salivation in the parotid, and submandibular and sublingual glands.

We measured each of the stimulated salivations defined by three frequencies (89 Hz = 1.5 V, 114 Hz = 2.0 V and 180 Hz = 3.3 V) with the interval time of 10 min. Subjects (21) gave informed consent to the procedures: the belly of the masseter muscles was stimulated for 3 min, and examinations carried out at 10-min intervals. In particular, the stimulated part of the belly of masseter muscles accorded with part of the parotid gland. Ratios of resting to stimulated saliva were analysed, namely, ratios indicating over 100%, are regarded as the effective gland by vibration. This result showed that about 95% (20/21) of subjects were affected by any one of three kinds of frequencies in vibrotactile stimulation. Thus, increased saliva with the vibration stimuli (adequate frequency) appears to be produced by the parotid gland. However, increased salivation in each gland showed various patterns: the right and/or left side of parotid and/or submandibular and sublingual glands are larger or smaller, as shown in Figure 5. Namely, only salivation in the right or left side of the parotid gland is increased (Figure 6a), only that in the right or left side of the submandibular and sublingual glands is increased (Figure 6b) and that in the all glands is increased (Figure 6c), following various vibrotactile stimulation.

**Figure 7:**
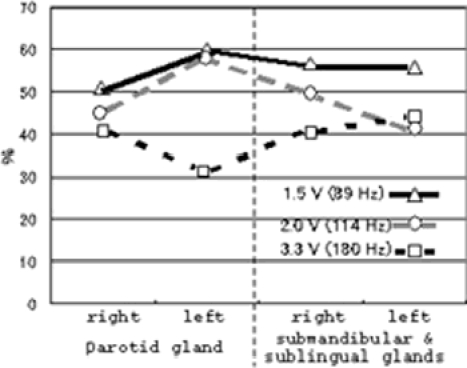
Effects in each gland of each frequency of vibrotactail stimulation.

**Figure 8:**
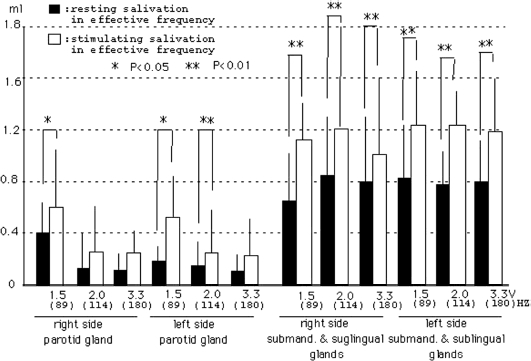
The relation between resting and stimulating saliva in the effective vibrotactail. Closed column: values of resting saliva shown by the effective vibrotactail. Open column: values of stimulating saliva in the effective vibrotactail. The relationship between these data carried out test with Paired *t*-test (*p < 0.05, **p < 0.01).

Furthermore, we examined the increased salivation depending on the frequency in each gland, as shown in Figure 7. In glands affected by each frequency, the parotid gland in the right side showed increase rates of 50%, 45% and 40% in 89 Hz (1.5 V), 114 Hz (2.0 V) and 180 Hz (3.3 V) of each subject, and the parotid gland in the left side showed increase rates of 60%, 60% and 30%, respectively. The submandibular and sublingual glands in the right side of each subject showed increase rates of 55%, 50% and 45%, and the submandibular and sublingual glands in the left side showed increase rates of 50%, 40% and 40%, respectively. In particular, subjects with the 89 Hz of vibrotactile stimulation (1.5 V) are the most effective in producing the salivation, as shown in Figure 7. Furthermore, we examined the relation between resting saliva and stimulated saliva in each gland of each subject during the effective variation. Furthermore, we made a comparison between the resting and stimulated salivation rates (3 min) in effective frequency, as shown in Figure 8. The results suggested that increased salivation was clearly 1.5 V (89 Hz) frequency in the parotid glands, and all frequencies in the submandibular and sublingual glands. Although subjects of increased vibration salivation of 114 and 180 Hz in submandibular and sublingual glands are much less common than that of 89 Hz from data in Figures 7 and 8, increased rates (3 min) are larger than those in the parotid glands.

We examined the effective increased salivation in the right and left sides, and in the parotid and submandibular and sublingual glands with each vibration. However, a unified view of glands in the increased salivation against various vibrations was not obtained.

## Discussion

### The large variation in each gland and in each subject

In the resting and stimulated saliva, rates (3 min) of averages (Avg.) and standard deviations (SD) are almost the same quantities in the right and left sides of glands with the same name, as shown in Figure 4. Although we generally disagree about the quantity of salivation with the average in each gland every so often in salivation of human, we must always take into consideration the large variation in each gland, in each subject and on either the right or left side of the same gland, as shown in Table I.

### Why did salivation increase with vibration stimuli?

Yrjama and Vanharanta (1994) reported that disco-graphically painful discs always produced painless feeling in the vibration examination. Furthermore, Burdette and Gale (1988) reported that tonic masticatory muscle activity might be effective in the care of myofascial pain-dysfunction patients. These facts assume that peripheral stimuli provided by vibration arrive at the central nerves (in the spinal cord and brain stem) and that these effects were exercised by the somatosensory information. Thus, we attempted to develop a vibration apparatus for effective rehabilitation of orofacial muscles. However, vibration stimuli on the bilateral belly masseter muscles with the apparatus provided increased salivary secretion in many subjects. Thus, we examined whether the increased salivation depending on the frequency was a fact or not.

We are interested in TVR (tonic vibration reflex) as a way of providing mechanical stimuli that is delivered to the orofacial muscles. In particular, masseter muscles have muscle spindles and are one of the principal closing muscles. Furthermore, the headphone headset of device designed by us, can simultaneously stimulate the bilateral belly of the masseter muscles. There are many reports (Desmedt et al. 1975; Desmedt and Godaux 1980; Clark et al. 1981; Grassi et al. 1993; Takata et al. 1996) about the effective frequency produced by the activation of muscle spindles or Aα-efferent fibres in masseter muscles. As their effective frequency showed 80–180 Hz, we tried to provided three vibration stimuli, 89 Hz (1.5 V), 114 Hz (2.0 V) and 180 Hz (3.3 V).

The increased salivation in either gland produced by either frequency was seen in 96% of subjects (22/23). In Figure 6, we showed the effect of increased salivation in each gland against each frequency. The vibration of 89 Hz (1.5 V) indicated increased rates of over 50% effect of subjects with increased salivation in the parotid gland of the right and left sides, and the submandibular and sublingual glands in the right and left sides. Namely, over 50% of subjects showed increased salivation in any one of each gland. Although the vibration of 114 Hz (2.0 V) indicated over 50% effect of subjects with increased salivation in the parotid gland of the left side, other glands (the parotid in the left side, and submandibular and sublingual glands in the right and left sides) showed below 50%. On the other hand, in the vibration of 180 Hz (3.3 V) indicated no effects of over 50%.

Why did salivation increase with the 89 Hz vibrotactile stimulation? We think that the vibration produces effective activation against the muscle spindles of the masseter muscles. In particular, the muscle spindles of the masseter muscles show the activation of tonic vibration reflex (TVR) with the 80–100 Hz vibrations (Desmedt and Godaux 1980). The parotid gland on the belly of masseter muscles will produce the salivary secretion with the activation and/or contraction of the muscles through muscle spindles. However, why did salivation from the submandibular and sublingual glands increase with the 89 Hz vibrotactile stimulation? We assume that vibration accompanied by the bone conduction, provides activation of the group of the suprahyoid muscles and produces increased salivation in the submandibular and sublingual glands. On the other hand, although we did not examine the increased blood flow in the masseter muscles depending on the vibration, we think it indicates increased salivation due to the increased metabolism. We will next examine this at around 80 Hz vibrotactile stimulation in our future research.

Although there are various mechanoreceptors in the facial skin, the four principal ones are Meissner’s corpuscles, Merkel disk receptors, Pacinial corpus-cles and Ruffini endings. The Merkel disk receptor has a small, highly localized receptive field, whereas the Ruffini endings have a large field with a central zone of maximal sensitivity (Gardner et al. 2000). Depending on their location, individual Ruffini endings are excited by stretch of the skin in specific directions. They are slowly adapting receptors. Meissner’s corpuscles on the fingertips have receptive fields averaging 2–3 mm in diameter, while receptive fields on the palm average 10 mm in diameter. The receptive field of Pacinian corpuscles covers larger continuous surfaces, but has a central zone of maximal sensitivity located directly above the receptor. They are rapidly adapting receptors (Gardner et al. 2000).

In particular, vibration is the sensation produced by sinusoidal oscillation of objects placed against the skin. The vibratory frequency is signalled by the frequency of action potentials fired by the sensory nerves, and individual mechanoreceptors differ in their threshold sensitivity to vibration. For example, Merkel disk receptors are most responsive to extremely low frequencies (5–15 Hz), Meissner corpuscles are response at 20–50 Hz, and Pacinian corpuscles are at 60–400 Hz (at 250 Hz they detect vibration as small as 1 μm, but at 30 Hz require stimuli with much larger amplitudes) (Gardner et al. 2000). Namely 89 Hz provided the most effective salivation, and this may be evoked by Pacinian corpuscles. Furthermore, mechanoreceptors’ stimuli conducted by vibration in the face, may be excited by the Merkek disk and Meissner’s corpuscles receptors through the mucosa in the oral cavity.

**Figure 9:**
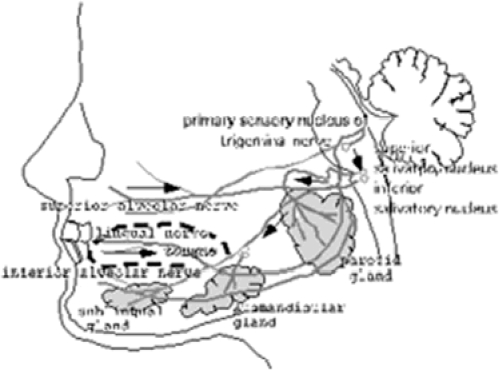
Schema of salivation. Arrows in the schema show information flows: somatosensory information evoked by mechanoreceptive stimuli in the oral cavity, arrive at the superior and inferior salivary nucleus, and these information are provided to the parotid, submandibular and sublingual glands by the impetus.

On the other hand, why did the vibration on the face produce an increase in the salivation of salivary glands? We think that these mechanoreceptors may be related to the reflex arc for the salivation via parasympathetic nerves. We assumed the existence of the salivary reflex with reflex arc among mechanoreceptors, trigeminal sensory nerves, trigeminal sensory complex nuclei inferior and superior salivary nuclei, facial and glossopharyngeal nerves, and parotid, submandibular and sublingual glands. In other words, people feel the increased salivation during chewing of food in the oral cavity. Furthermore, during dental pain we feel the increased salivation. In particular, we think that there is a possibility of salivation producing by mechanical stimuli of food in the oral cavity and facial skin. We think that increased salivation with vibration stimuli may be produced by almost the same effects as food stimuli in the facial skin and oral cavity on chewing, as shown in Figure 9. We would also point out the decrease in salivation by vibrotactile stimulation in comparison with the resting salivation. In particular, some glands may act as the excitation to some vibrotactile stimuli, and other vibrotactile stimuli may act as the inhibition. The findings suggest that each gland maybe have a unique frequency (eigenfrequency).
